# Transmission of Zika virus by dendritic cell subsets in skin and vaginal mucosa

**DOI:** 10.3389/fimmu.2023.1125565

**Published:** 2023-03-06

**Authors:** Julia Eder, Esther Zijlstra-Willems, Gerrit Koen, Neeltje A. Kootstra, Katja C. Wolthers, Teunis B. Geijtenbeek

**Affiliations:** ^1^ Department of Experimental Immunology, Amsterdam UMC location University of Amsterdam, Amsterdam, Netherlands; ^2^ Amsterdam Institute for Infection and Immunity, Amsterdam, Netherlands; ^3^ Department of Medical Microbiology and Infection Prevention, Amsterdam UMC location University of Amsterdam, Amsterdam, Netherlands

**Keywords:** Zika virus, vaginal mucosa, Langerhans cells, virus transmission, viral dissemination

## Abstract

Zika virus is a member of the Flaviviridae family that has caused recent outbreaks associated with neurological malformations. Transmission of Zika virus occurs primarily *via* mosquito bite but also *via* sexual contact. Dendritic cells (DCs) and Langerhans cells (LCs) are important antigen presenting cells in skin and vaginal mucosa and paramount to induce antiviral immunity. To date, little is known about the first cells targeted by Zika virus in these tissues as well as subsequent dissemination of the virus to other target cells. We therefore investigated the role of DCs and LCs in Zika virus infection. Human monocyte derived DCs (moDCs) were isolated from blood and primary immature LCs were obtained from human skin and vaginal explants. Zika virus exposure to moDCs but not skin and vaginal LCs induced Type I Interferon responses. Zika virus efficiently infected moDCs but neither epidermal nor vaginal LCs became infected. Infection of a human full skin model showed that DC-SIGN expressing dermal DCs are preferentially infected over langerin+ LCs. Notably, not only moDCs but also skin and vaginal LCs efficiently transmitted Zika virus to target cells. Transmission by LCs was independent of direct infection of LCs. These data suggest that DCs and LCs are among the first target cells for Zika virus not only in the skin but also the genital tract. The role of vaginal LCs in dissemination of Zika virus from the vaginal mucosa further emphasizes the threat of sexual transmission and supports the investigation of prophylaxes that go beyond mosquito control.

## Introduction

Zika virus is a mosquito-borne flavivirus containing a single-stranded positive RNA (1). It belongs to the Flaviviridae family and is closely related to dengue virus (DENV) and West Nile virus (WNV) (2). People infected with Zika virus are mostly asymptomatic or experience mild symptoms including fever, arthralgia and a maculopapular rash (3, 4). However, Zika virus outbreaks have been associated with severe neuropathologies including microcephaly, congenital deafness and impaired vision, termed Congenital Zika Syndrome in neonates infected *in utero* and Guillain-Barré syndrome in adults (5–8). Consequently, the World Health Organization (WHO) labeled in 2016 the Zika virus pandemic in South America a public health emergency (9). Zika virus is the only Flavivirus that passes the maternal-placental barrier (10) and infects placental cells that include villous stromal macrophages, i.e. Hofbauer cells, and placental trophoblasts (11–14). While the exact transmission route for Zika virus over the maternal-fetal barrier is still unclear, physical disruption, and transcytosis have been described *in vitro* (15) and placental damage observed in animal models (16–18). Zika virus has further been identified in amniotic fluid and tissues of the developing fetus in infected pregnant women (19, 20). Importantly, Zika virus is also transmitted sexually (21–26). RNA of Zika virus is present in seminal fluid (27, 28) as well as in vaginal fluid (26). Monocytes and Dendritic cells (DCs) are targets for Zika virus infection and might be involved in dissemination of Zika virus (29–33), as these cells are found in barrier tissues like skin and mucosal surfaces (32, 34–36). Amongst the DC subsets susceptible to Zika virus are monocyte-derived DCs (moDCs) (33, 37). Zika virus infection of moDCs leads to productive virus replication and secretion (29, 33, 36, 38).

DC-SIGN, a C-type lectin receptor (CLR) expressed on DC subsets, facilitates Zika virus binding and infection *in vitro* (39). DC-SIGN is expressed on DCs and macrophages (40, 41) and enables infection of viruses like HIV-1 and dengue virus (40, 42).

Langerhans cells (LCs), a subset of DCs, are located in the outmost layer of the skin and genital tract (43, 44) where they are one of the first cells to encounter and sense viruses (45–48). However, LCs are also targets for virus infections like HIV-1 (49, 50). In healthy tissue, LCs restrict HIV-1 infection by capture through CLR langerin receptor and subsequent degradation in Birbeck granules (51–53). The role of LCs in the skin and genital tract during Zika virus infection is still largely unclear.

Here we have investigated the role of DC subsets in skin and vaginal mucosa in Zika virus infection. We observed that Zika virus efficiently infected primary DCs *via* DC-SIGN, and blocking of the receptor inhibited infection as well as transmission. LCs isolated from human skin or vagina were resistant to Zika virus infection, however LCs efficiently transmitted Zika virus to susceptible target cells. These observations suggest a role for DCs and LCs in the dissemination of Zika virus from site of infection throughout the body.

## Results

### moDCs become activated by Zika virus leading to type I interferon responses

**Figure 1:**
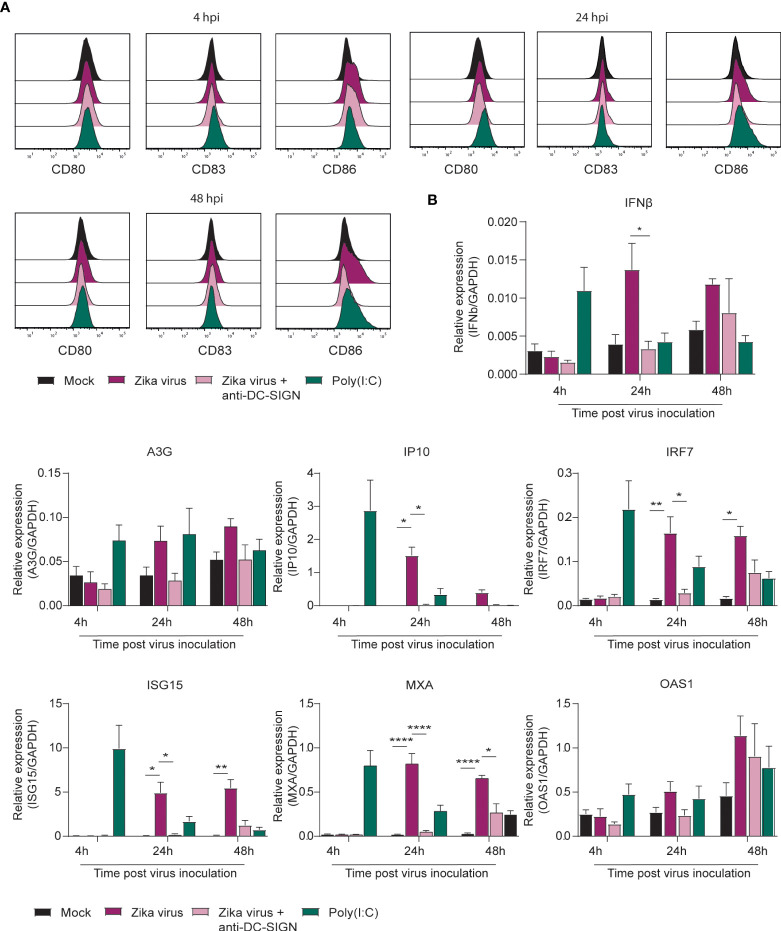
Zika virus induces activation and interferon responses in monocyte derived DCs **(A)** Monocyte derived DCs (moDCs) were pre-incubated with Poly(I:C) or a blocking antibody against DC-SIGN (AZN-D1) before Zika virus was added at a concentration of 850 TCID/ml. Cells were fixed after either 4 hours, 24 hours or 48 hours and expression of activation and maturation markers was measured *via* flow cytometry (1 representative donor out of 4 individual donors measured in monoplo). **(B)** (moDCs) pre-incubated with Poly(I:C) (10 µg/ml) or AZN-D1 (20 µg/ml) and subsequently infected with Zika virus (850 TCID/ml) were lysed and expression of IFNβ and interferon stimulated genes (ISG) was measured on PCR after 4 hours, 24 hours and 48 hours respectively. Data information: Data show the mean values and error bars are the SEM. Statistical analysis was performed using **(B)** ordinary one-way ANOVA with Tukey multiple comparison test. *P ≤ 0.05, **P ≤ 0.01, ****P ≤ 0.0001 (n=4 donors measured in monoplo). hpi: hours post inoculation.

Type I IFNs and interferon stimulated genes (ISGs) are important antiviral responses (54–57). Here we investigated whether Zika virus (primary human isolate, Asian lineage) activates moDCs and induces type I IFN responses. We observed upregulation of the co-stimulatory molecules CD80 and CD86 at 24 and 48 hours post inoculation (hpi) (**Figure 1A**; **Supplementary Figure 1A**). Antibodies against CLR DC-SIGN blocked Zika virus-induced upregulation of the co-stimulatory markers CD80 and CD86 by Zika virus (**Figure 1A**; **Supplemenraty Figure 1A**). Expression of the maturation marker CD83 was not induced by Zika virus, whereas Poly(I:C), a TLR-3 antagonist, induced low expression of CD83 (**Figure 1A** for single donor representation and the pooled data in **Supplementary Figure 1A**). Upregulation of CD80 and CD86 but not CD83 suggests that the cells are not fully matured but activated and primed for antigen presentation. Next we investigated whether Zika virus inoculation of moDCs induces type I IFN responses. Zika virus induced upregulation of IFN beta (IFNβ) at 24 hpi and 48 hpi (**Figure 1B**; **Supplementary Figure 1A**). Moreover, ISGs Apobec3G, IP10, IRF7 and MXA were induced highest at 24 hpi whereas ISG15 and OAS1 peaked later at 48 hpi (**Figure 1B**). Antibodies against DC-SIGN blocked induction of IFNβ, IP10, MXA, ISG15, IRF7, A3G and OAS1, albeit not significantly for A3G and OAS1 at 48 hpi (**Figure 1B**). Blocking viral replication by the viral polymerase inhibitor 7-Deaza-2’-C-Methyladenosine (7DMA) abrogated IFNβ and ISG transcription to a similar extent as blocking infection by the blocking antibody against DC-SIGN (**Supplementary Figure 1B**). Moreover, heat inactivated Zika virus [inactivated as described previously (58)] did not lead to induction of IFNβ or ISGs in moDCs. These data indicate that moDCs sense Zika virus *via* DC-SIGN, leading to moderate DC maturation and induction of antiviral immunity.

### Immature skin LCs are not activated by Zika virus

**Figure 2:**
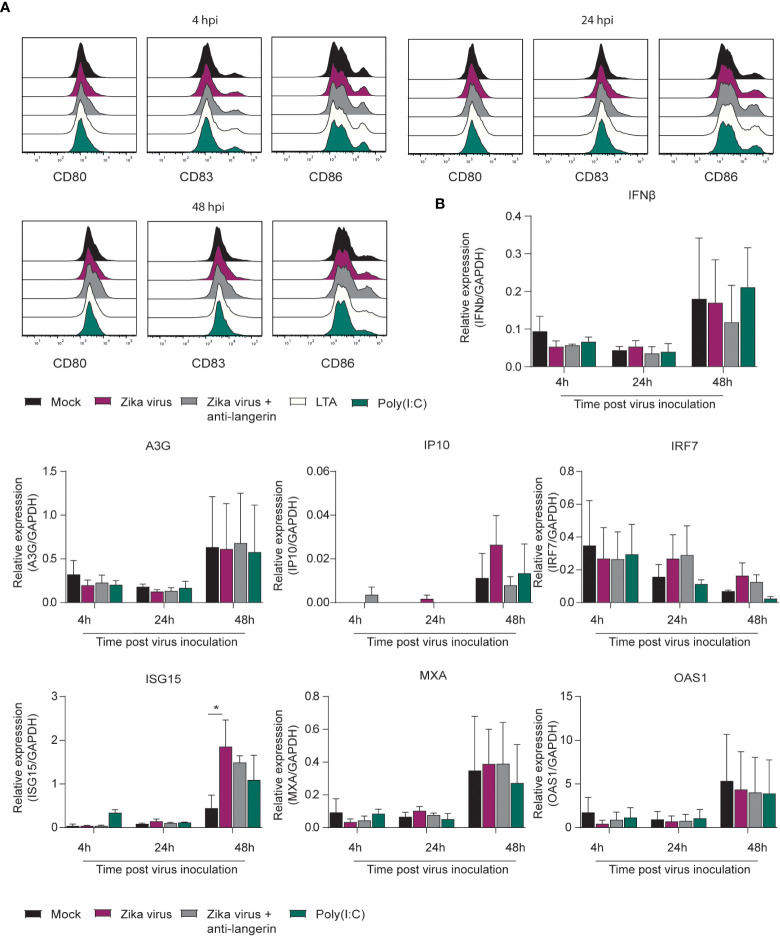
Zika virus does not induce activation or interferon responses in skin derived LCs **(A)** Immature Langerhans cells (LCs) isolated form epidermal skin grafts were pre-incubated with either Poly(I:C) (10 ng/ml), LTA (10 ng/ml), (1µM) or a blocking antibody against langerin (10E2, 20 µg/ml) before Zika virus was added at a concentration of (850 TCID/ml). Cells were fixed after either 4 hours, 24 hours or 48 hours and expression of activation and maturation markers was measured *via* flow cytometry (1 representative out of 3 individual donors measured in monoplo). **(B)** Immature LCs pre-incubated with Poly(I:C) or AZN-D1 and subsequently infected with Zika virus (850 TCID/ml) were lysed and expression of IFNβ and interferon stimulated genes (ISG) was measured on PCR after 4 hours, 24 hours and 48 hours respectively. Data information: Data show the mean values and error bars are the SEM. Statistical analysis was performed using **(B)** ordinary two-way ANOVA with Tukey multiple comparison test. *P ≤ 0.05, (n=3 donors measured in monoplo). hpi: hours post inoculation.

Immature LCs were isolated by CD1a selection from human skin. Immature skin LCs do not express DC-SIGN but are instead characterized by expressing the CLR langerin (59–61). Following exposure of LCs to Zika virus, LC activation and type I IFN responses was determined. Zika virus did neither induce CD80, CD83 nor CD86 in immature LCs (**Figure 2A**; **Supplementary Figure 1B**). Moreover, we did not observe maturation after stimulation with TLR antagonists Poly(I:C) and LTA. However, Zika virus induced ISG15 and IP10, whereas IFNβ and other ISGs were not detected (**Figure 2B**). Blocking CLR langerin did not affect LC activation nor type I IFN responses. These data suggest that LCs are not activated by Zika virus.

### DC-SIGN is involved in Zika virus binding and transmission

**Figure 3:**
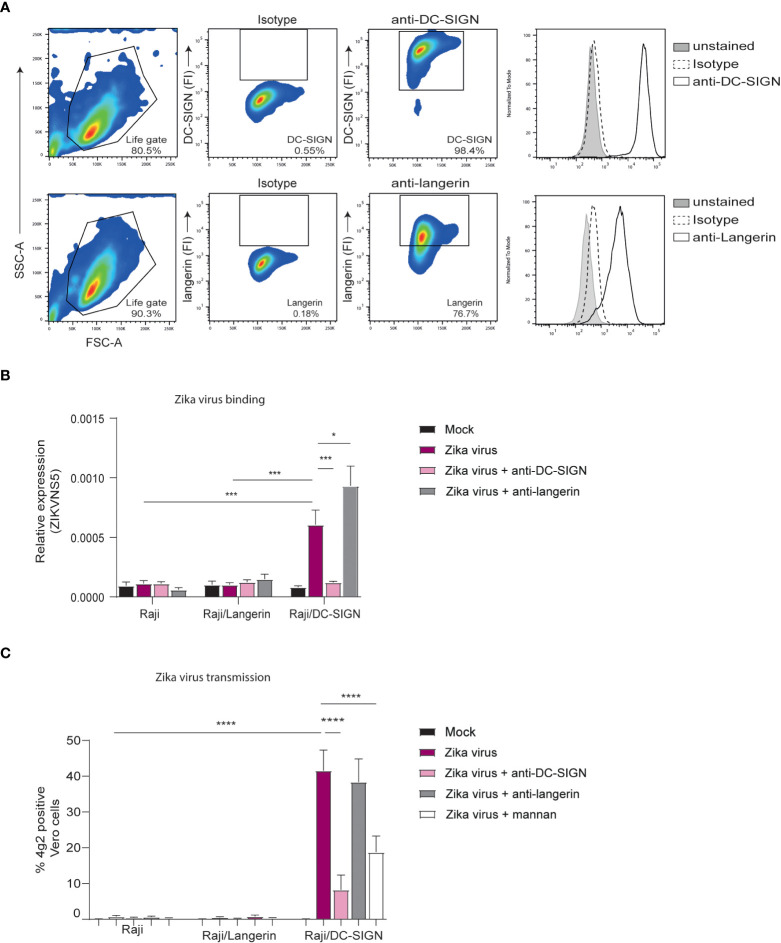
Zika virus binds to DC-SIGN but not langerin for transmission **(A)** Raji cells expressing either DC-SIGN or langerin as measured by flow cytometry (n=1 representative donor). **(B)** Raji, Raji/SIGN and Raji/Langerin cells were exposed to Zika virus (175 TCID/ml) for 4 hours before measuring binding of Zika virus NS5 protein. Quantification of viral RNA was measured by quantitative real-time PCR. Additionally, cells were pre-incubated with either an anti-DC-SIGN antibody (AZN-D1, 20 µg/ml) or an anti-langerin antibody (10E2, 20 µg/ml) prior to virus inoculation. **(C)** Raji cells were inoculated with Zika virus (35 TCID/ml) in presence or absence of AZN-D1, 10E2 or mannan for 4 hours. After washing the Raji, the cells were co-cultured with Vero cells for another 2 to 3 days to determine viral transmission. Zika virus infection of Vero cells was measured by flow cytometry (4g2 Flavivirus envelope protein). Data information: Data show the mean values and error bars are the SEM. Statistical analysis was performed using **(B)** ordinary two-way ANOVA with Tukey’s multiple-comparison test. *P ≤ 0.05, ***P ≤ 0.001 (n=4 experiments measured in monoplo). **(C)** ordinary two-way ANOVA with Tukey’s multiple-comparison test. ****P ≤ 0.0001 (n=4 experiments measured in triplicate).

DC-SIGN and langerin are both important C-type lectins and known attachment receptor for various viruses (62). Importantly, DC-SIGN has already been shown to bind Zika virus *in vitro* (39, 63). Here we investigate whether DC-SIGN and langerin interact with Zika virus. To this end, we employed a Raji cell line selectively expressing either DC-SIGN or langerin (**Figure 3A**). DC-SIGN expressing Raji cells efficiently bound Zika virus in contrast to parental or langerin-expressing Raji cells. Moreover, binding by DC-SIGN-Raji was blocked by antibodies against DC-SIGN but not by isotype antibodies (**Figure 3B**). Next we investigated whether Zika virus binding to DC-SIGN facilitates viral transmission. Notably, DC-SIGN-Raji incubated with Zika virus for 4 hours successfully transmitted Zika virus to target cells in contrast to Langerin-Raji (**Figure 3C**). Mannan, a carbohydrate used to block CLRs, or antibodies against DC-SIGN but not the isotypes control significantly reduced Zika virus transmission by DC-SIGN Raji, suggesting that DC-SIGN captures and transmits Zika virus (**Figure 3C**). These data strongly suggest that DC-SIGN, in contrast to langerin, is involved in Zika virus binding and transmission.

### DC-SIGN+ primary moDCs are susceptible to Zika virus infection and transmission

**Figure 4:**
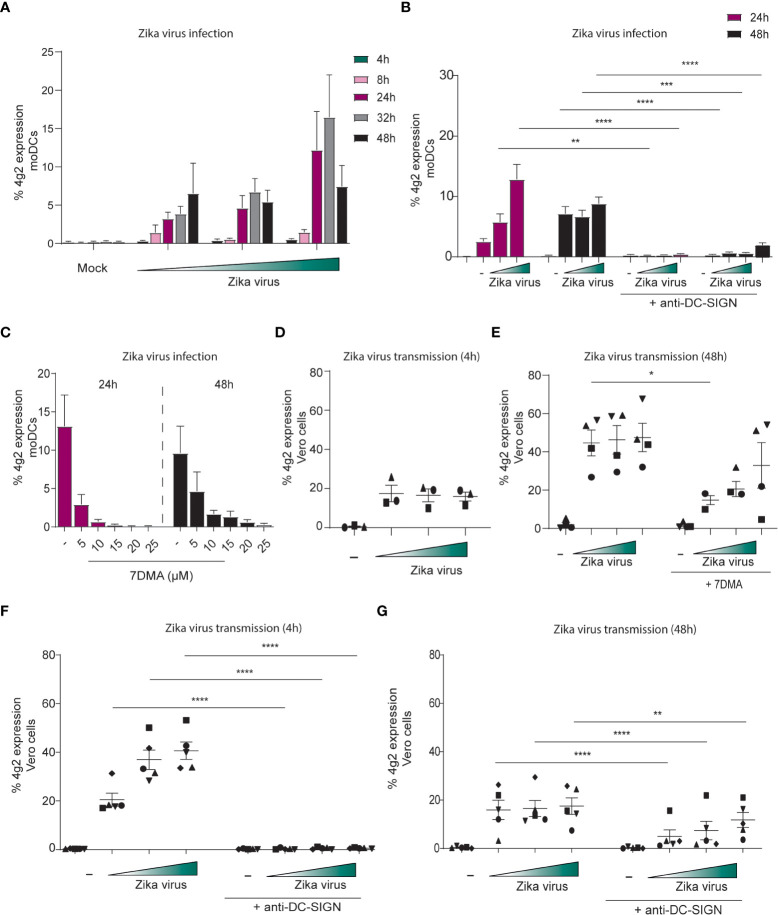
DC-SIGN positive moDCs are susceptible to Zika virus infection and can transmit the virus to target cells **(A)** moDCs were inoculated with Zika virus for different time points (4, 8, 24, 32 and 48 hours) before infection was measured by flow cytometry (4g2 Flavivirus protein) (n=4 donors measured in duplicates). **(B)** moDCs in presence of absence of an anti-DC-SIGN antibody (AZN-D1, 20 µg/ml) were exposed to Zika virus (750 TCID/ml) for either 24 or 48 hours. Infection was measured by flow cytometry (4g2 Flavivirus envelope protein). **(C)** The Zika virus replication inhibitor 7DMA was added to moDCs in different concentrations (5- 25 µM) prior to Zika virus (750 TCID/ml) inoculation. Zika virus infection (4g2 Flavivirus protein) was determined after 24 or 4 8 hours. (n=2 donors measured in duplicate). **(D)** moDCs were incubated with Zika virus for 4 hours at 37°C, extensively washed and co-cultured with Vero cells for another 3 days. (n=3 donors measured in triplicates) **(E)** moDCs pre-incubated with 7DMA were exposed to Zika virus (750 TCID/ml) for 48 hours at 37°C. After washing, the cells were co-cultured with Vero cells. Transmission by moDCs to Vero cells was determined flow cytometry (4g2 Flavivirus envelope protein). **(F, G)** moDCs in the presence or absence of anti-DC-SIGN antibody(AZN-D1, 20 µg/ml) were incubated with Zika virus for either 4 hours **(F)** or 48 hours at 37°C **(G)**. After washing, moDCs were co-cultured with Vero cells and Vero infection was measured by flow cytometry (4g2 Flavivirus envelope protein) **(G)** n=5 measured in triplicates. Data information: Data show the mean values and error bars are the SEM. **(B)** ordinary two-way ANOVA with Tukey’s multiple-comparison test. **P ≤ 0.01, ***P ≤ 0.001, ****P ≤ 0.0001, (n=4 donors measured in duplicate). **(E)** ordinary two-way ANOVA with Tukey’s multiple-comparison test. *P ≤ 0.05, (n=4 donors measured in triplicate). **(F)** ordinary two-way ANOVA with Tukey’s multiple-comparison test. ****P ≤ 0.0001, (n=5 donors measured in triplicate). **(G)** ordinary two-way ANOVA with Tukey’s multiple-comparison test. **P ≤ 0.01, ****P ≤ 0.0001, (n=5 donors measured in triplicate). Zika virus concentrations are 375, 750 or 2250 TCID/ml. moDC: monocyte derived moDCs.

To determine whether DCs are susceptible to Zika virus infection, moDCs were inoculated with increasing concentrations of Zika virus and expression of viral proteins was measured at different time points after inoculation by flow cytometry. Zika virus infection of moDCs was detected after 8 hpi and increased up to 48 hpi compared to mock infected moDCs (**Figure 4A**). Infection levels increased over time and with higher virus concentration. Cell viability remained constant for 48 hours (**Supplementary Figure 2B**). moDCs highly express DC-SIGN (**Supplementray Figure 2C**) and antibodies against DC-SIGN blocked infection of moDCs without influencing cell viability (**Figure 4B**; **Supplementary Figure 2D**). Soluble mannan, also inhibited Zika virus infection of moDCs (**Supplementary Figure 2E**). The TAM receptors Tyro3 and AXL, candidate receptors for Zika virus infection (64), are also expressed on moDCs. Similarly, skin derived LCs highly express Tyro3 but instead of AXL they express MerTK (**Supplementary Figure 3A**). Moreover, viral polymerase inhibitor 7DMA blocked moDC infection in a concentration dependent manner 24 hpi and 48 hpi (**Figure 4C**). These data indicate that Zika virus productively infects DCs.

We next investigated whether moDCs transmit Zika virus to Zika virus-permissive Vero cells. moDCs were exposed to Zika virus for 4 hours and were co-cultured with Vero cells after washing. Vero cell infection was determined by flow cytometry. Notably, Vero cells became infected by Zika virus (**Figure 4D**). As moDCs were not productively infected after 4 hours, these data suggest that moDCs transmit virus independent of infection. Next we infected moDCs for 48 hours and after washing co-cultured moDCs with Vero cells. Zika virus-infected moDCs efficiently transmitted Zika virus to Vero cells, and transmission was inhibited by the replication inhibitor 7DMA (**Figure 4E**). 7DMA inhibition decreased but did not block viral transmission at higher inoculum, suggesting that part of the transmission is replication independent. Antibodies against DC-SIGN completely blocked transmission of moDCs treated with Zika virus for 4 or 48 hours (**Figures 4F, G**). These data suggest that Zika virus efficiently infects primary moDCs and that DC-SIGN is involved in infection and transmission of Zika virus by DCs.

### Epidermal Langerhans cells transmit Zika virus

**Figure 5:**
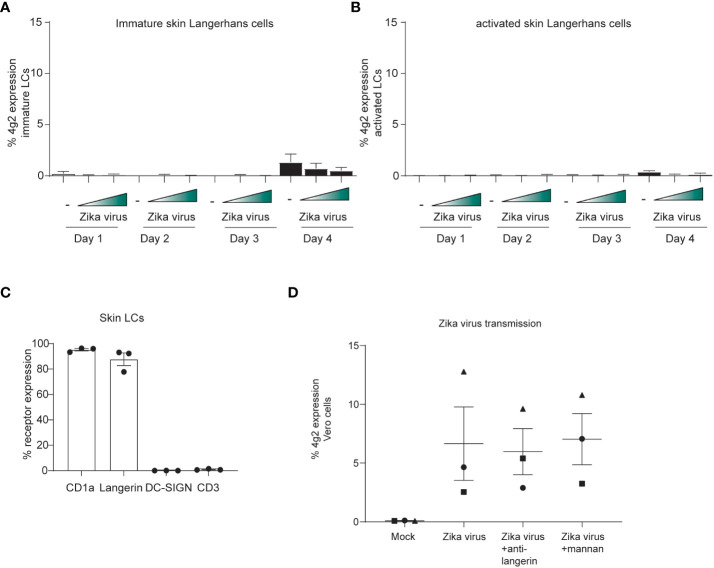
Skin LCs do not become infected by Zika virus but are involved in viral dissemination through transmission. **(A)** Immature LCs were isolated from human skin and exposed to Zika virus (750 or 2250 TCID) for up to 4 days. Zika virus infection (4g2 Flavivirus envelope protein) was measured by flow cytometry. (n=4 donors 2 measured in triplicates 2 in monoplo). **(B)** Activated LCs migrated from epidermal skin sheets after 3 days were exposed to Zika virus (750 or 2250 TCID) for up to 4 days and infection (4g2 Flavivirus envelope protein) was measured by flow cytometry (n=3 donors measured in triplicates). **(C)** Immature LCs isolated from skin were stained for langerin, CD1a, CD3 and DC-SIGN and expression was measured by flow cytometry (n=3 individual donors). **(D)** Immature LCs were inoculated with Zika virus (850 TCID/ml) for 4 hours. After thoroughly washing the LCs, they were co-cultured with Vero cells for 3 days. Prior to the addition of Zika virus, skin LCs were incubated with either anti-langerin antibody (10E2, 20 µg/ml) or mannan (100 µg/ml). (n=3 donors measured in triplicates). Data information: Data show the mean values and error bars are the SEMLC: Langerhans cell.

Next, immature LCs from human skin were incubated with Zika virus and infection was followed over time. We did not observe any Zika virus-positive LCs at different time points (**Figure 5A**). Moreover, activated LCs isolated after migration from skin sheets, were not infected by Zika virus (**Figure 5B**). These data suggest that skin-derived LCs are not permissive to ZIVK infection. Importantly, immature skin LCs do not express DC-SIGN but express the CLR langerin and high levels of CD1a (**Figure 5C**). We next incubated immature LCs with Zika virus for 4 hours and co-cultured them with Vero cells. Notably, Vero cells became infected by Zika virus after co-culture with LCs (**Figure 5D**) indicating that skin-derived LCs transmit Zika virus. Transmission was not abrogated by antibodies against langerin, suggesting that langerin is not involved in Zika virus infection nor transmission.

### DC subsets in the skin are targeted by Zika virus

**Figure 6:**
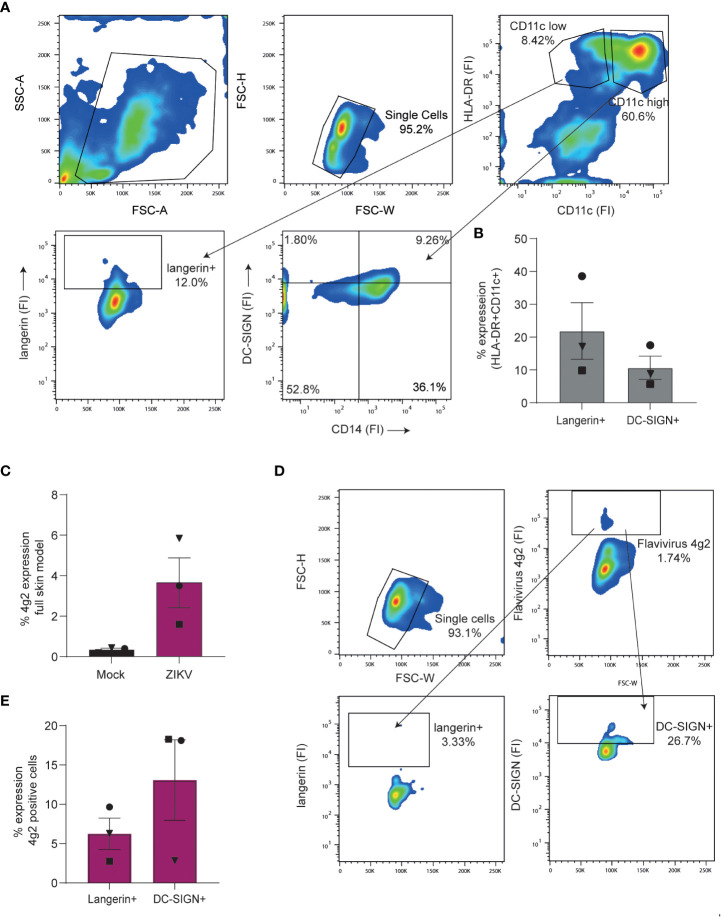
Dendritic cell subsets in a full skin explant model are susceptible to Zika virus **(A, B)** Full skin explants consisting of a dermal and epidermal layer were incubated for 3 days at 37°C during which skin cells crawled out of the tissue and into the medium. **(A)** Expression of cell surface markers of cells retrieved from full skin explants as measured by flow cytometry. Cells were stained with antibodies against HLA-DR, CD11c, CD14, langerin and DC-SIGN to identify and separate DC subsets. (1 representative of n=4 donors measured in triplicates). **(B)** Pooled data of dermal cells being either CD11c high/HLA-DR+ and DC-SIGN+ or CD11c low/HLA-DR+ and expressing langerin (n=4 donors in triplicates). **(C–E)** Full skin explants were exposed to Zika virus (1100 TCID/ml) for 3 days before infection was measured by flow cytometry (n=3 donors measured in triplicates). **(C)** Zika virus infection (4g2 Flavivirus envelope protein) of full skin explants was determined by flow cytometry. **(D)** Zika virus positive cells were stained for DC-SIGN and langerin to further determine cell subset infection. Infection of either langerin or DC-SIGN+ cells was measured by anti-4G2 Flavivirus envelope protein. Data information: data show the mean values and error bars are the SEM.

We next exposed full skin explants to Zika virus and determined both phenotype and infection of migrated cells. Phenotyping was based on previously described dermal cell markers (65). After 3 days, migrated cells consisted of CD11c high/HLA-DR^+^ DCs that could further be divided into CD14^+^DC-SIGN^-^ and CD14^+^DC-SIGN^+^ DC subsets. Moreover, we identified a CD11c low/HLA-DR^+^ cell population expressing langerin, suggesting that these are migrated LCs (**Figures 6A, B**). Notably, we observed Zika virus infection in the migrated cells (**Figure 6C**). Further phenotyping revealed that DC-SIGN^+^ dermal cells were more readily infected by Zika virus than langerin^+^ LCs (**Figures 6D, E**). Moreover, Zika virus infected cells highly expressed CD11c (**Supplementary Figure 3B**), indicating that these are dermal DCs. Thus our data support a role for DC-SIGN expressing cells as targets for Zika virus infection in skin.

### Vaginal LCs transmit Zika virus to target cells

**Figure 7:**
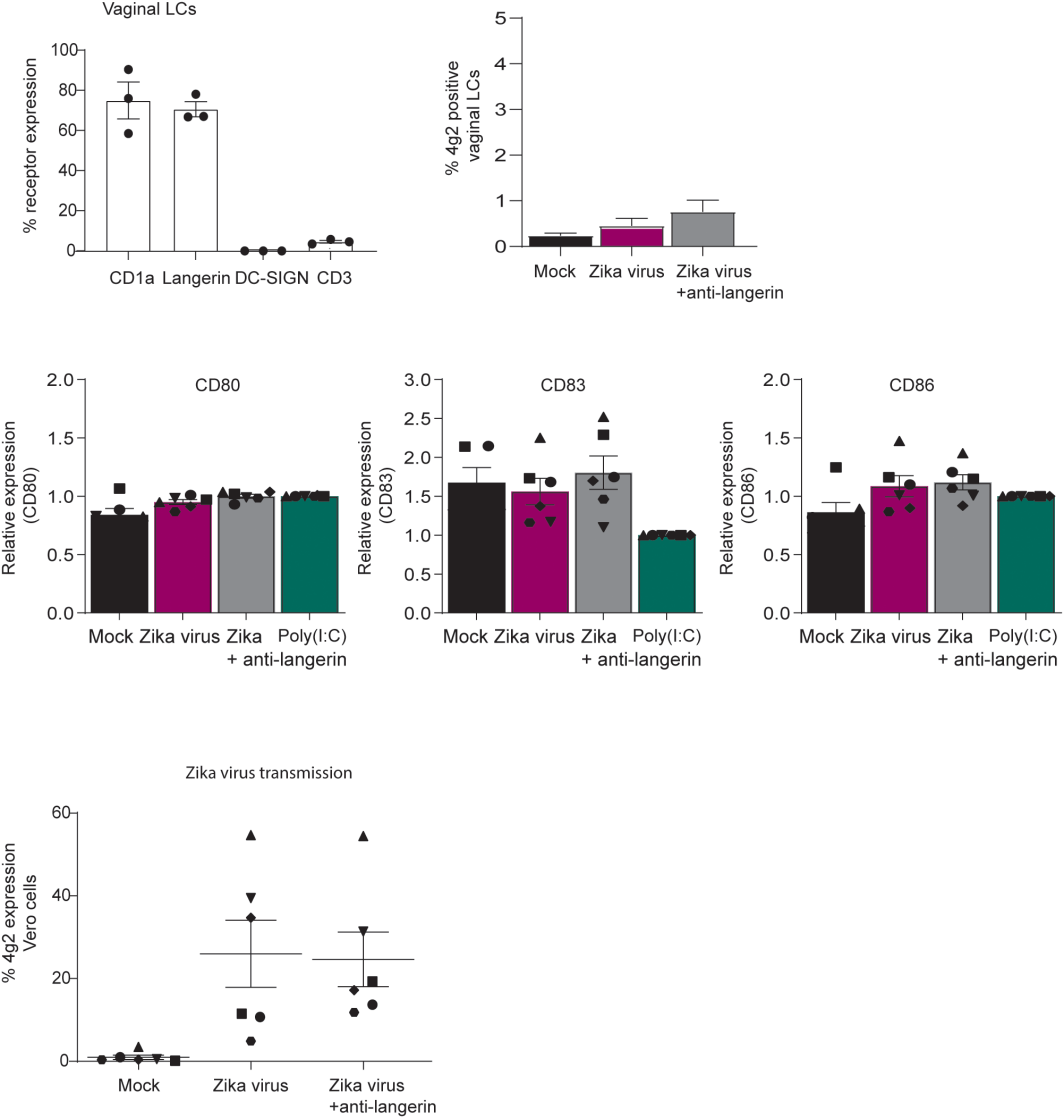
Vaginal LCs from mucosal tissues transmit Zika virus to target cells independent of infection. **(A)** Immature LCs isolated from vaginal mucosa were characterized for their high expression of CD1a and langerin and lack of DC-SIGN and CD3, as measured by flow cytometry (n=3 individual virginal LC donors). **(B)** Vaginal immature LCs were exposed to Zika virus (850 TCID/ml) for 3 days. Zika virus infection (4g2 Flavivirus envelope protein) was measured by flow cytometry. (n=5 individual donors measured in monoplo). **(C)** Vaginal immature LCs were pre-incubated with Poly(I:C) (10 ng/ml) or an antibody against langerin (10E2, 20 µg/ml) for 1 hour prior to Zika virus (850 TCID/ml) inoculation. After 24 hours, the expression of activation and maturation markers CD80, CD83 and CD86 were measured by flow cytometry (n=6 donors measured in monoplo). **(D)** Vaginal immature LCs were inoculated with Zika (850 TCID/ml) virus for 4 hours before the cells were washed extensively and transmitted to Vero cells for co-culture of another 3 days. Prior to Zika virus exposure, the cells were incubated with anti-langerin antibody 10E2 (20 µg/ml). Vero cell infection was measured by anti-4g2 Flavivirus envelope protein on flow cytometry. **(D)** n=6 donors measured in monoplo). Data information: Data show the mean values and error bars are the SEM.

Zika virus can be transmitted sexually (21–26), and therefore vaginal mucosa is an important tissue for viral entry. We isolated vaginal LCs from vaginal mucosa obtained after prolapse surgery and compared these with skin LCs. Vaginal LCs expressed high levels of CD1a and langerin (**Figure 7A**) (48). Vaginal LCs are highly similar to skin derived LCs in their expression of CD1a and langerin but lack of DC-SIGN (**Figure 7A**). Immature vaginal LCs were not infected by Zika virus after 3 days (**Figure 7B**). Similar to what we observed for skin derived LCs, Zika virus did not induce maturation of vaginal LCs (**Figure 7C**).

Notably, vaginal LCs incubated with Zika virus for 4 hours transmitted Zika virus to target cells independent of langerin (**Figure 7D**). These results suggest that LCs in vaginal mucosa transmit Zika virus to target cells and thereby contribute to viral dissemination in the genital tract, regardless of infection.

## Discussion

Zika virus continues to circulate endemically in many American and Asian regions (66–68), highlighting the need for better understanding of the virus. Understanding the mechanism of Zika virus infection and transmission are paramount for better prevention and treatment in the future. The (re)emergence of several Flavivirus species in the Americas and Europe (including Zika virus, WNV and YFV) over the past few years suggests that Zika virus could expand to hitherto unaffected areas (69), and due to climate change conditions more favorable for mosquito breeding and virus replication may persist in these areas (70).

Little is known about the primary target cells that facilitate Zika virus infection after mosquito bites or sexual contact. Here, we show that different DC subsets are involved in dissemination of Zika virus. Zika virus infected monocyte-derived DC as well as DC subsets in skin, in contrast to epidermal and vaginal LCs. Interestingly, both moDCs and LCs transmitted Zika virus to target cells. Our data strongly suggest that skin and vaginal LCs as well as moDCs are important in viral dissemination of Zika virus.

Zika virus stimulation of moDCs lead to a slight increase in the expression of activation markers CD80 and CD86 whereas CD83 was not affected by Zika virus. Moreover, we observed that Zika virus induced type I IFN responses in moDCs after 24 hours, which suggests that infection of moDCs leads to the induction of type I IFN responses similar as observed for dengue virus infection (71, 72). As we do not observe induction of type I IFN or ISG at early time points, our data suggest that viral replication is required to induce type I IFN responses and that replication intermediates are sensed. Inhibiting Zika virus replication with the viral polymerase inhibitor 7DMA blocked IFNβ transcription 24 hours post inoculation, further indicating that Zika virus infection is important for type I IFN responses. Antibodies against DC-SIGN blocked type I IFN responses as well as infection to a similar extent as the replication inhibitor, supporting that viral replication is a prerequisite for DC activation. Finally, heat-inactivated Zika virus did not induce any type I IFN responses, suggesting that sensing of viral material alone is not sufficient. These data are in line with previous reports that support replicative Zika virus infection of moDCs and induction of type I IFN responses as well as maturation (31, 38). Interestingly, Bowen et al. suggested that Zika virus blocks IFNβ production leading to low maturation and limited type I IFN responses in moDCs following ZIKV infection (29). Several studies have shown that Zika virus evades antiviral IFN responses. Zika virus NS5 interferes with IFN signaling by inducing degradation of STAT2 proteins (73–75), whereas induction of lipid metabolism has also been suggested to suppress antiviral responses (33). Although we observed that productive infection of moDCs leads to type I IFN responses, we cannot exclude that these responses are limited by Zika virus and might be responsible for the low DC maturation. Moreover, while Zika viruses of the Asian and African lineage do not induce strong maturation and activation in moDCs, IFN induction is nonetheless observed for both lineages (31), confirming that Zika virus replication does not completely abrogate type I IFN responses. Further studies are required to understand the functional consequences on induction of antiviral innate and adaptive immunity to Zika virus by infected DCs.

Conversely, Zika virus did not activate skin derived LCs. Moreover, neither TLR antagonist induced a strong activation, suggesting these cells are difficult to activate. We have shown previously that migration of LCs from epidermal explants induces strong maturation (76). However, we observe some induction of ISG15 after 4 hours and 24 hours, indicating that these cells are functional.

While we observed strong binding of Zika virus to cells overexpressing DC-SIGN, we did not observe binding to those expressing langerin. Zika virus binding to DC-SIGN has been described before in different cell lines (39, 77, 78) but involvement of other CLRs is still largely unclear (79). Our data suggest that DC-SIGN is important for Zika virus infection of moDCs isolated form the blood and migrated DCs from a full skin explant model, whereas langerin is not.

Importantly, DC-SIGN expressing cells did not only get infected by Zika virus but also efficiently transmitted Zika virus to target cells, suggesting a role for DC-SIGN in viral dissemination. These data support the notion that Zika virus hijacks DC-SIGN expressing cells for viral dissemination in a process similar to what has been observed previously for other viruses like HIV-1 and recently also SARS-CoV-2 (80–82). DCs can transmit viruses like HIV-1 *via* two different pathways: *cis* and *trans*-transmission (83). For *cis*-transmission, DCs are productively infected and new virions are transmitted to target cells (84), whereas trans-infection refers to the replication independent transfer of virions (83). We observed cis-transmission of Zika virus after 48 hours of co-culture with moDCs. Interestingly, we also observed Zika virus transmission by moDCs already after 4 hours of co-culture, where we did not observe infection yet, indicating that Zika virus is transmitted independent of infection. DC-SIGN inhibition abrogated viral dissemination by the cis as well as trans pathway, suggesting that viral capture *via* DC-SIGN is crucial for Zika virus infection and transfer. However, block of transmission was strongest during trans-infection whereas the protective effect started to wane with longer moDC incubation periods, suggesting that DC-SIGN inhibition is transient. While moDCs efficiently transmitted Zika virus both after 4 hours and 48 hours of incubation, we observed variability in transmission efficacy that are likely attributed to differences in the human primary moDC donors.

Importantly, our data show that DC-SIGN on primary moDCs and skin-derived dermal cells is involved in Zika virus infection and transmission. DC-SIGN expressing cells can be found in many Zika virus target tissues including genital mucosa and the placenta (85, 86), rendering DC-SIGN a promising receptor for preventative approaches against Zika virus.

Importantly, our data describe infection and dissemination of DC-SIGN expressing moDCs with a Zika virus strain of Asian lineage. This strain is the one that was first linked to neurological pathogenicity (87) and the strain that is closest related to samples isolated from the outbreak in Brazil (88). While there are differences observed in Vero cell and insect cell susceptibility of different Zika virus strains, no such differences were observed in primary human moDCs (31). However, Zika virus from the Asian lineage show higher infection rate in both moDCs and macrophages than a historical African strain (38), indicating that moDCs are more susceptible to Asian strain Zika viruses.

LCs, like DCs, migrate to lymph nodes, but are also closely related to macrophages and repopulate locally independent of blood circulation (43, 89). We observed no Zika virus induced activation of co-stimulatory molecules in epidermal LCs. Interestingly, while LCs did not produce IFNβ or most ISGs, there was significant upregulation of IP10 and ISG15 after 48 hours of Zika virus inoculation. Lack of clear IFNβ induction might be due to our finding that LCs do not become infected by Zika virus. Neither immature nor activated LCs isolated from skin and vaginal mucosa were infected by Zika virus. Moreover, in a full skin explant model, DC-SIGN expressing cells were preferentially infected over langerin expressing LCs. Notably, we observed that both skin and vaginal LCs transmitted Zika virus to target cells. As we did not observe infection in either of the two cell types, the mode of transmission is likely through trans-infection. These data suggest that skin and vaginal LCs might be involved in Zika virus dissemination. The main route of Zika virus transmission is through mosquito bite. We therefore used skin explants from human donor tissue and could verify that DC-SIGN+ cells in the skin become infected with Zika virus. In contrast, langerin expressing cells did not become well infected, supporting their resistance to infection in the skin. However, we observed low levels of langerin expressing cells becoming infected by Zika virus. This is likely contributed to environmental factors present in *in situ* skin explants that are missing in isolated single cell suspension.

Importantly, the female reproductive tract harbors different LC subsets in the epithelial layer of the vagina (90, 91). LCs we isolated from vaginal mucosa are similar to skin LCs in their co-expression of langerin and CD1a and similarly did not get infected but efficiently transmitted Zika virus to target cells.

Our data strongly suggest that after sexual transmission, ZIKV migrates from the genital tract with the help of LCs. As langerin was not involved in transmission, further studies are required to identify the receptor(s) involved in the transmission of Zika virus by vaginal LCs. In conclusion, DC-SIGN renders primary human DC subsets susceptible to Zika virus infection while also facilitating transmission of infectious virus. Using primary human skin and vaginal mucosa we have uncovered an important role for LCs in the capture and transmission of Zika virus, thereby contributing to viral dissemination and infection. Further investigation into the LC receptors responsible for Zika virus transmission might lead to better understanding and prevention of sexual transmission of Zika virus.

## Materials and methods

### Study approval

This study was performed according to the Amsterdam University Medical Centers, location AMC, Medical Ethics Committee guidelines. This study, including the tissue harvesting procedures, was conducted in accordance with the ethical principles set out in the declaration of Helsinki and was approved by the institutional review board of the Amsterdam University Medical Centers and the Ethics Advisory Body of the Sanquin Blood Supply Foundation (Amsterdam, Netherlands). All research was performed in accordance with appropriate guidelines and regulations.

### Isolation of monocyte derived Dendritic cells

CD14+ monocytes were obtained from buffy coats of healthy volunteer donors (Sanquin blood bank) and differentiated into monocyte derived DCs as described previously (92). In short, first PBMCs were isolated with lymphoprep. Subsequently, monocytes were collected after percoll gradient steps. Monocytes were cultured in RPMI medium supplemented with 10% FCS, l‐glutamine (2 mM, Lonza), penicillin and streptomycin (100 U/mL and 100 μg/mL, respectively, Thermo Fisher) in the presence of GM‐CSF (800 U/mL, Invitrogen) and IL‐4 (500 U/mL Invitrogen) at 37°C, 5% CO2 for 6 days to obtain monocyte‐derived DCs.

### Isolation of Langerhans cells from epidermis

Skin LCs were isolated from human epidermal sheets obtained from healthy donors undergoing corrective plastic surgery. Epidermal sheets were prepared as described previously (51, 76). Briefly, skin-grafts consisting of epidermis and dermis were obtained using a dermatome (Zimmer Biomet, Indiana USA). Upon overnight incubation with Dispase II (1 U/mL, Roche Diagnostics), epidermal sheets were separated from dermis, washed and either directly subjected to enzymatic treatment with trypsin and DNAse to obtain immature skin LCs or alternatively, cultured in IMDM (Thermo Fischer Scientific, USA) supplemented with 10% FCS, gentamycine (20 μg/mL, Centrafarm, Netherlands), pencilline/streptomycin (10 U/mL and 10 μg/mL, respectively; Invitrogen) for 3 days after to harvest activated LCs. Immature as well as activated skin LCs were purified by ficoll gradient (Axis-shield). Immature skin LCs were further subjugated to CD1a magnetic cell separation (MACS, Miltenyi Biotec). Purity of LCs was routinely verified by flow cytometry using antibodies directed against CD207 (langerin) and CD1a.

### Full skin ex vivo sheets

Ex vivo sheets of human skin were obtained from healthy donors undergoing corrective surgery. The top two layers of the skin were prepared as described previously (51). After retrieval of a thin layer (thickness at 12pt) containing both the epidermis and dermis, biopsies with a diameter of 8 mm (Kai medical) were prepared. The biopsies were placed on 500 uL of IMDM (Thermo Fischer Scientific, USA) supplemented with 10% FCS, gentamycine (20 μg/mL, Centrafarm, Netherlands), pencilline/streptomycin (10 U/mL and 10 μg/mL, respectively; Invitrogen). Zika virus was added at a concentration of 1000 TCID/ml after which the sheets were left at 37°C. After 3 days, the sheets were removed and the medium containing the emigrated cells was subjected to further analyses.

### Isolation of Langerhans cells from vaginal mucosa

Human vaginal tissue was collected from women undergoing prolapse surgery where excessive vaginal tissue was removed from the anterior or posterior vaginal wall. Surplus stroma was removed from mucosal sheets dissected until a thin layer of submucosa remained and tissue was cut into strips of 5-7 mm. Vaginal tissue strips were incubated overnight at 4°C in complete medium (Iscoves Modified Dulbecco’s Medium (IMDM) of Thermo Fischer Science with L-glutamine 100 mmol/L, 10% FCS, 2500 U/mL penicillin, and 2500 mg/mL streptomycin) supplemented with Dispase II (3 U/mL, Roche Diagnostics). After incubation, the epithelial layer and lamina propria were mechanically split by the use of tweezers. Vaginal epithelial sheets were extensively washed in PBS after which was proceeded with immature LC isolation.

Immature vaginal LCs were obtained after mucosal sheets were cut in small pieces using surgical scissors and incubated for 30 minutes in PBS containing trypsin (0,05%, BD Biosciences) and DNAase I (20 U/mL, Roche Applied Science) to obtain a single cell suspension. Further vaginal LC purification was achieved by ficoll gradient centrifugation (Axis-shield) and CD1a magnetic cell separation (MACS, Miltenyi Biotec).

### Cell lines

The African monkey Vero cells (ATCC^®^ CCL-81™) were maintained in MEM with Earle’s Salts (Capricorn Scientific, Ebsdorfergrund, Germany) supplemented with 10% fetal calf serum (FCS), L-glutamine and penicillin/streptomycin (10 μg/mL) as well as non-essential amino acids (NEAA). Culture was maintained at 37C with 5% CO_2_. The human B cells, Raji (ATCC^®^ CCL-86™) as well as Raji transfectants stably expressing human DC-SIGN or human langerin created by electroporation (40) were cultured in RPMI 1640 medium (Gibco Life Technologies, Gaithersburg, Md.) containing 10% fetal calf serum (FCS), penicillin/streptomycin (10 μg/mL). The expression of DC-SIGN and langerin was regularly checked *via* FACS analysis.

### Zika virus production

The following reagent was obtained from the European Virus Archive goes global: Zika virus, strain H/PF/2013 (clinical isolate, Asian lineage), French Polynesia 2013 with Ref-SKU: 001v-EVA1545 and GenBank number KJ776791.2. Vero cells (ATCC^®^ CCL-81™) were inoculated with the Zika virus isolate and used for reproduction of virus stocks. Formation of cytopathic effect (CPE) was closely monitored and after observing a CPE of 4+, supernatant containing the virus was filtered (0.2 µm) and stored at -80C.

### Tetrazolium dye colorimetric cell viability (MTT) assay

Viral titers were determined by tissue culture infectious dose (TCID50) on Vero cells by MTT assay. In brief, Vero cells were seeded in a 96 well plate at a cell density of 10.000 cells per well. After 24 hours, cells were inoculated with a 5-fold serial dilution of Zika virus. Cell cytotoxicity was measured 72 hours after infection. MTT solution was added to Vero cells and incubated for 2 hours at 37°C. After removing the MTT solution, MTT solvent containing 4 mM HCL and 1% Nonidet P-40 (NP40) in isopropanol was added to the cells. Homogenous solution was measured at optical density between 580 nm and 655 nm. Loss of MTT staining as determined by spectrometer is indicative of CPE caused by Zika virus infection. The virus titer was determined as TCID50/mL and calculated based on the Reed Muench method (93).

### Reagents

The following reagents were used: to inhibit Zika virus replication, the viral polymerase inhibitor 7-Deaza-2’-C-Methyladenosine (7DMA) (#ND08351, Carbosynth) as described in (94). Cells were stimulated with lipopolysaccharide (LPS) Salmonella enterica serotype typhimurium (10 ng/mL, Sigma) or Poly(I:C) and Invitrogen (10 μg/mL, *In vivo*gen).

### Zika virus infection

Isolated primary cells as well as cell lines and full skin explants were inoculated with Zika virus at different TCID/ml concentrations. Viral infection was determined after two to three days post inoculation *via* flow cytometry staining. Zika virus infection was measured with antibodies against either a Flavivirus envelope protein. Viral binding was determined by RT-PCR after 4 hours. Zika virus was heat-inactivated for 60 min at 60°C as described by (58).

### Cell maturation

Monocyte derived DCs, immature skin and immature vaginal LCs were exposed to either Poly(I:C) or LTA (both Invitrogen) at a concentration of 10 µg/ml. Additionally, skin derived LCs were exposed to 1 µM of Motolimod (VTX124 2337, MedChemExpress). Simultaneously, cells were inoculated with Zika virus at a concentration of 850 TCID/ml with or without the presence of a C-type lection inhibitor (AZN-D1 for DCs, 10E2 for LCs) or mannan. After 24 hours at 37°C, cells were either fixed to continuing with FACS analysis or lysed for subsequent PCR analysis.

### Flow cytometry

Cells were fixed with paraformaldehyde (PFA) and treated with either BSA (extracellular staining) or BSA/Saponin to measure intracellular staining. The following antibodies were used to detect Zika virus: anti-Flavivirus 4g2 mouse IgG2a (NovusBio), anti-Flavivirus 4g2 monoclonal rabbit (Absolute Antibody), anti-Zika virus monoclonal Rabbit (Genetex).

All other antibodies were anti-human: DC-SIGN mouse IgG1 (AZN-D1), anti-langerin mouse IgG1 (10E2) both in house made, PE conjugated CD207 (langerin), APC conjugated CD1a (BD Biosciences), CD86-FITC (BD Pharmingen), CD80-PE (BD Pharmingen), CD83-APC (BD Pharmingen), DC-SIGN-FITC (R&D systems), CD3-APC/Fire750 (Biolegend), CD11c-APC (Biolegend), PEcy7-HLA-DR (BD Pharmingen), APCcy7-CD14 (BD Biosciences), APCcy7-CD11c (Biolegend),APC-AXL (Thermofisher, PE-MerTK (Thermofisher) and anti-Tyro3 (Thermofisher). For secondary detection the following antibodies were used: AF488-conjugated goat anti-mouse IgG2a (Invitrogen), AF647-conjugated donkey anti-rabbit (Thermofisher), FITC-conjugated goat-anti-mouse IgM (Invitrogen), AF488-conjugated donkey anti-rabbit (Thermofisher).

Flow cytometric analyses were performed on a BD FACS Canto II (BD Biosciences) and data was analysed using FlowJo V10 software (TreeStar).

### Zika virus binding

To determine Zika virus binding to C-type lectins DC-SIGN and langerin, Raji cells were seeded at a density of 100.000 cells in 100 μl. Cells were kept in FCS free medium to increase receptor expression prior to virus exposure. Zika virus was added at a concentration of 175 TCID/ml and the cells were left at 4°C for 4 hours. Subsequently, cells were washed extensively to remove any unbound virus before lysis with AVL buffer. RNA was isolated with the QIAamp Viral RNA Mini Kit (Qiagen) according to the manufacturer’s protocol.

### Transmission assays and co-culture

Raji cells were exposed to 35 TCID/ml Zika virus for 4 hours. DC-SIGN or langerin receptors were blocked prior to virus inoculation antibodies against AZN-D1 or 10E2 for 1 hour at 37°C. Primary DCs were exposed to ZIVK with 425, 850 or 2550 TCID/ml. Skin or vaginal immature LCs were exposed to 805 TCID/ml of Zika virus. Prior to virus inoculation, DC-SIGN or langerin receptors were blocked with antibodies against AZN-D1 or 10E2 respectively in certain conditions. Infection was determined after incubation with the virus for multiple days and assessed by flow cytometry. Additionally, primary cells were stimulated with LPS or Poly(I:C) for 24h before infection. Virus transmission to Vero target cells was determined by incubating Raji, DCs or LCs with Zika virus for either 4h or 48h. After, cells were washed extensively to remove unbound virus and subsequently co-cultured with Vero cells for 3 days. To assess Zika virus transmission, infection of Vero cells was measured by flow cytometry.

### RNA isolation and quantitative Real Time-PCR

Viral RNA in cells was isolated using the QIAamp Viral RNA Mini Kit (Qiagen) according to the manufacturers protocol. cDNA was subsequently synthesized with the M-MLV reverse-transcriptase kit (Promega). cDNA samples were diluted 1 in 5 before further application. Cellular mRNA of cells not exposed to virus was isolated with an mRNA Capture kit (Roche) and cDNA was synthesized with a reverse-transcriptase kit (Promega). PCR amplification for all targets was performed in the presence of SYBR green in a 7500 Fast Realtime PCR System (ABI). Specific primers were designed with Primer Express 2.0 (Applied Biosystems). Primer sequences used for mRNA expression were for gene product: GAPDH, forward primer (CCATGTTCGTCATGGGTGTG), reverse primer (GGTGCTAA GCAGTTGGTGGTG). For gene product Zika virus-NS5, forward primer (CTTGTGGCTGCTGCGGAGGTCA), reverse primer(AACACGCTAACAAAGCACTCGTGGTGGGAGCAAAACGGAACTT) as described previously (95). For gene product IFNb, forward primer (ACAGACTTACAGGTTACCTCCGAAAC), reverse primer (CATCTGCTGGTTGAAGAATGCTT); for OAS1, forward primer (TGCGCTCAGCTTCGTACTGA), reverse primer (GGTGGAGAACTCGCCCTCTT); APOBEC3G, forward primer (TTGAGCCTTGGAATAATCTGCC), reverse primer (TCGAGTGTCTGAGAATCTCCCC); MXA, forward primer (TTCAGCACCTGATGGCCTATC), reverse primer (GTACGTCTGGAGCATGAAGAACTG); IRF7, forward primer (GCTCCCCACGCTATACCATCTAC), reverse primer (GCCAGGGTTCCAGCTTCAC); IP10, forward primer (CGCTGTACCTGCATCAGCAT), reverse primer (CATCTCTTCTCACCCTTCTTTTTCA); for ISG15, forward primer (TTTGCCAGTACAGGAGCTTGTG), reverse primer (GGGTGATCTGCGCCTTCA). The normalized amount of target mRNA was calculated from the Ct values obtained for both target and household mRNA with the equation Nt = 2Ct (GAPDH) − Ct(target).

### Cell viability assay

MTT solution was added to Vero cells and incubated for 2 hours at 37°C. After removing the MTT solution, MTT solvent containing 4 mM HCL and 1% Nonidet P-40 (NP40) in isopropanol was added to the cells. Homogenous solution was measured at optical density between 580 nm and 655 nm. For cell viability check with the CellTiter-Glo^®^ Luminescent Cell Viability Assay (Promega), cells were mixed with the buffer in a 1:1 ratio. The cells were treated according to the manufacturers protocol and measured with a luminometer.

### Statistics

All results are presented as mean ± SEM and were analyzed by GraphPad Prism 8 software (GraphPad Software Inc.). A two-tailed, parametric Student’s *t*-test for unpaired observation, Mann-Whitney tests (differences between different donors, that were not normally distributed) was performed. For unpaired, non-parametric observations a one-way ANOVA or two-way ANOVA test with *post hoc* analysis (Tukey’s or Dunnet’s) were performed. Statistical significance was set at *P< 0.05, **P<0.01***P<0.001****P<0.0001.

## Data availability statement

The original contributions presented in the study are included in the article/**Supplementary Material**. Further inquiries can be directed to the corresponding author.

## Author contributions

JE conceived and designed experiments. JE and EZ-W performed the experiments, acquired data and analyzed data. JE, NK and TG interpreted data and contributed to scientific discussion. GK, NK and KW provided essential research materials. GK helped with experiments and data acquisition. JE and TG wrote the manuscript with input from all listed authors. TG perceived of the original study idea and was involved in all aspects of the study. All authors approved the final version of the manuscript. The corresponding author vouches for the completeness and accuracy of the data. All authors contributed to the article and approved the submitted version.
