# Supplementary figures and images for: Transmission of Zika virus by dendritic cell subsets in skin and vaginal mucosa
Source: Front Immunol. 2023 Mar 6;14:1125565. doi: 10.3389/fimmu.2023.1125565 (PMC10025456; doi:10.3389/fimmu.2023.1125565)

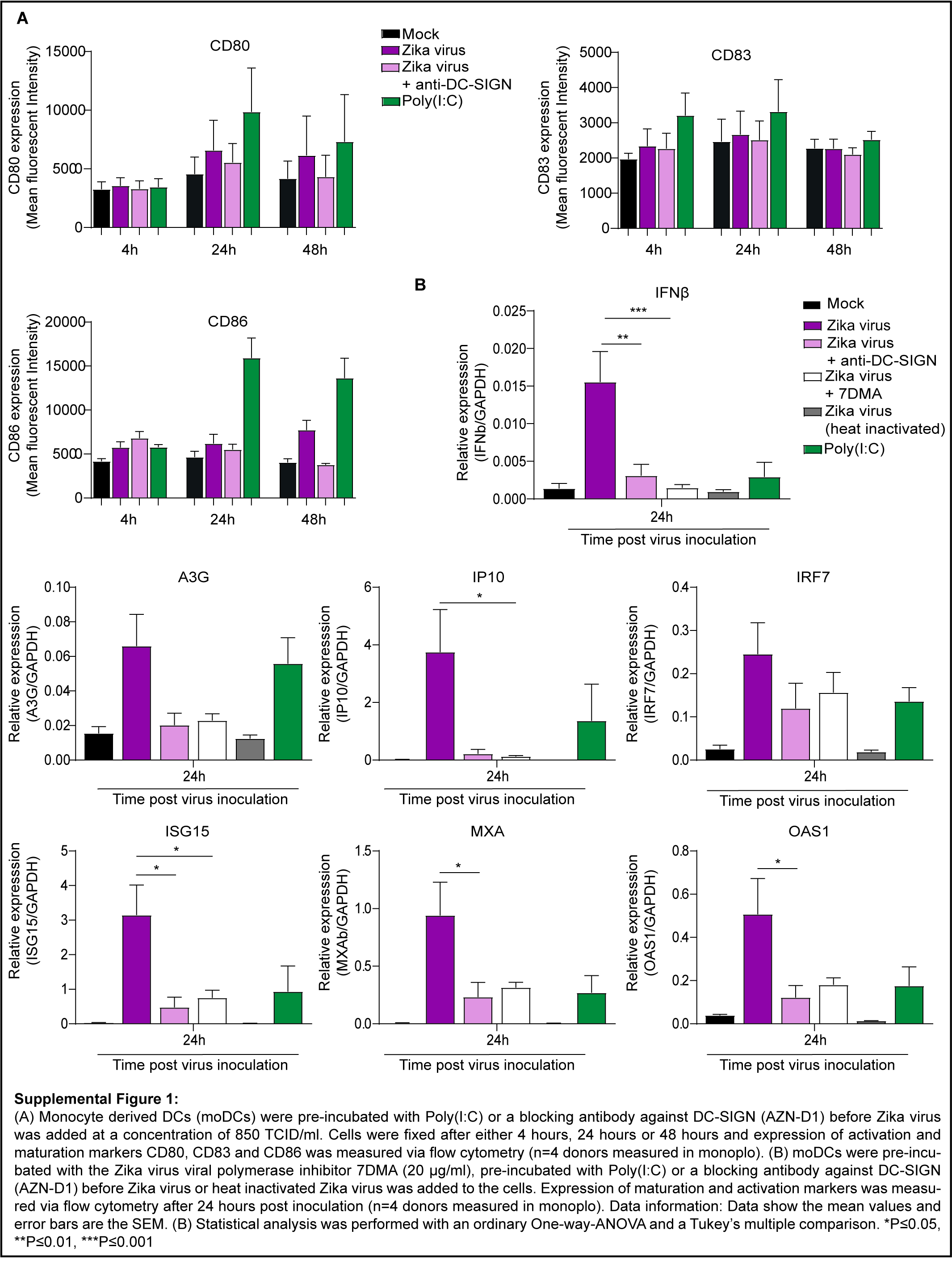

Supplement: Supplementary file 1 [file Image_1.tif]

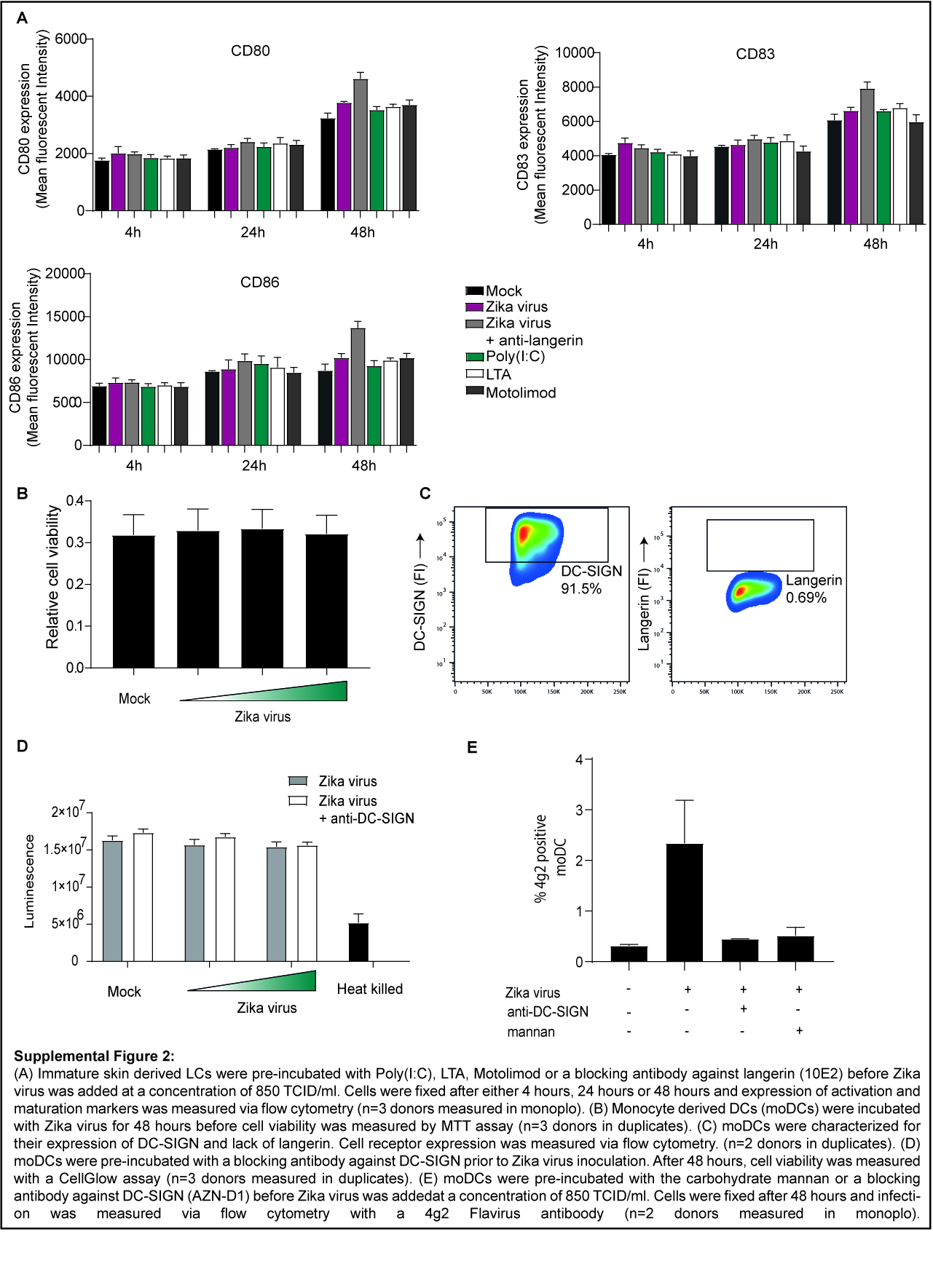

Supplement: Supplementary file 2 [file Image_2.tif]

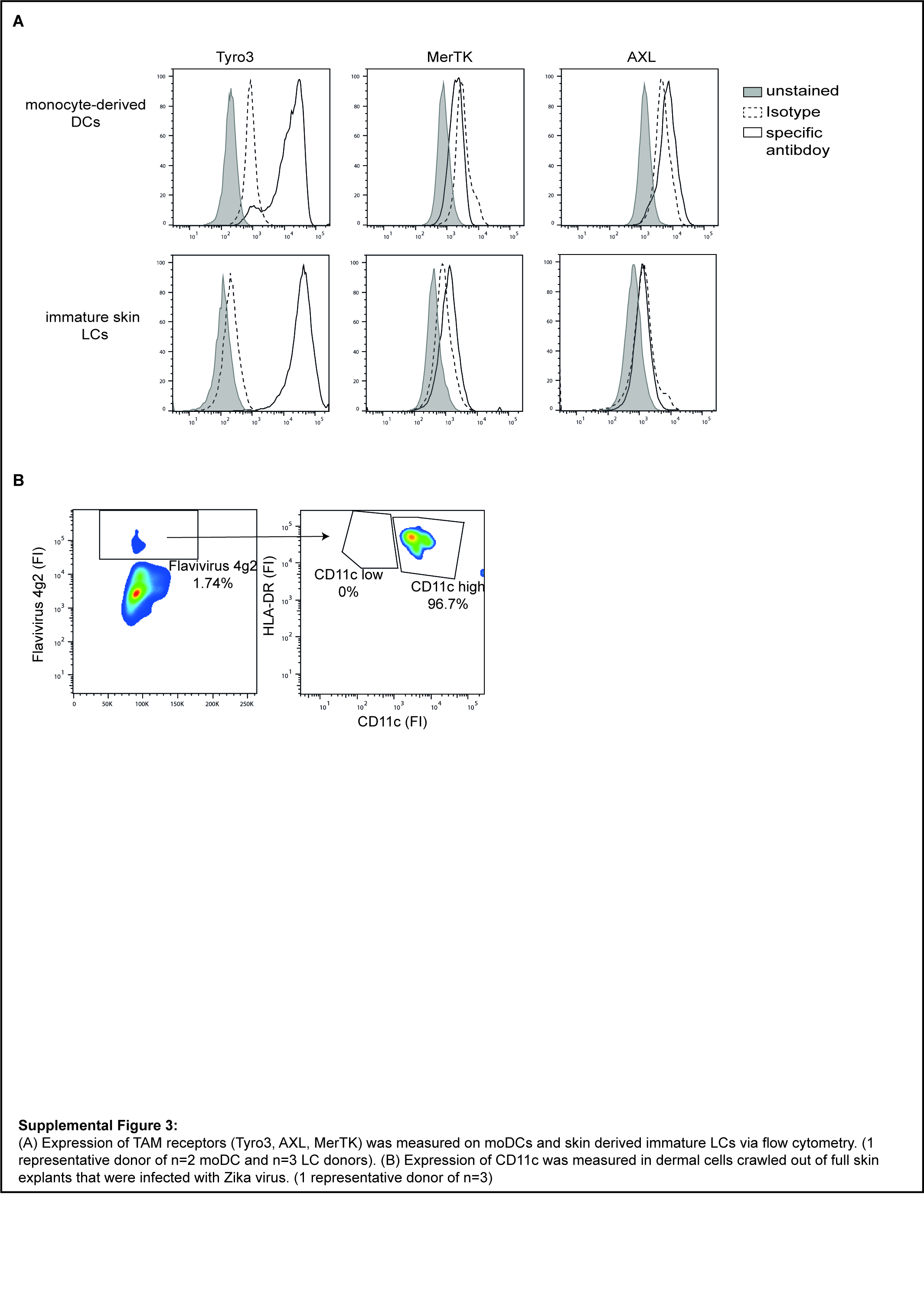

Supplement: Supplementary file 3 [file Image_3.tif]
